# Supplementary material for: Pyruvate Dehydrogenase Kinase 1 inhibition mediated oxidative phosphorylation enhancement in cartilage promotes osteoarthritis progression
Source: BMC Musculoskelet Disord. 2023 Jul 20;24:597. doi: 10.1186/s12891-023-06585-6 (PMC10357736; doi:10.1186/s12891-023-06585-6)
Supplement: Supplementary file 1 — Additional file 1. [file 12891_2023_6585_MOESM1_ESM.zip › BP.Gsea.1653623667859/GOBP_CELLULAR_RESPIRATION.html]

Details for gene set GOBP\_CELLULAR\_RESPIRATION[GSEA]

|  || Dataset | 55457\_collapsed\_to\_symbols.cls.cls #OA\_versus\_normal.cls.cls #OA\_versus\_normal\_repos |
| Phenotype | cls.cls#OA\_versus\_normal\_repos |
| Upregulated in class | OA |
| GeneSet | GOBP\_CELLULAR\_RESPIRATION |
| Enrichment Score (ES) | 0.4509358 |
| Normalized Enrichment Score (NES) | 2.320134 |
| Nominal p-value | 0.0 |
| FDR q-value | 7.48546E-5 |
| FWER p-Value | 0.001 |
Table: GSEA Results Summary

  

Fig 1: Enrichment plot: GOBP\_CELLULAR\_RESPIRATION      
 Profile of the Running ES Score & Positions of GeneSet Members on the Rank Ordered List

  

| SYMBOL | TITLE | RANK IN GENE LIST | RANK METRIC SCORE | RUNNING ES | CORE ENRICHMENT || 1 | UQCR10 | "ubiquinol-cytochrome c reductase, complex III subunit X [Source:HGNC Symbol;Acc:HGNC:30863]" | 53 | 0.987 | 0.0117 | Yes |
| 2 | NDUFA8 | NADH:ubiquinone oxidoreductase subunit A8 [Source:HGNC Symbol;Acc:HGNC:7692] | 67 | 0.961 | 0.0261 | Yes |
| 3 | DGUOK | deoxyguanosine kinase [Source:HGNC Symbol;Acc:HGNC:2858] | 96 | 0.917 | 0.0386 | Yes |
| 4 | PDHB | pyruvate dehydrogenase E1 subunit beta [Source:HGNC Symbol;Acc:HGNC:8808] | 149 | 0.862 | 0.0484 | Yes |
| 5 | NDUFS3 | NADH:ubiquinone oxidoreductase core subunit S3 [Source:HGNC Symbol;Acc:HGNC:7710] | 177 | 0.836 | 0.0597 | Yes |
| 6 | NDUFA1 | NADH:ubiquinone oxidoreductase subunit A1 [Source:HGNC Symbol;Acc:HGNC:7683] | 194 | 0.826 | 0.0717 | Yes |
| 7 | GHITM | growth hormone inducible transmembrane protein [Source:HGNC Symbol;Acc:HGNC:17281] | 213 | 0.809 | 0.0833 | Yes |
| 8 | PANK2 | pantothenate kinase 2 [Source:HGNC Symbol;Acc:HGNC:15894] | 220 | 0.805 | 0.0957 | Yes |
| 9 | NDUFA10 | NADH:ubiquinone oxidoreductase subunit A10 [Source:HGNC Symbol;Acc:HGNC:7684] | 243 | 0.788 | 0.1067 | Yes |
| 10 | NDUFB6 | NADH:ubiquinone oxidoreductase subunit B6 [Source:HGNC Symbol;Acc:HGNC:7701] | 262 | 0.779 | 0.1178 | Yes |
| 11 | MDH1 | malate dehydrogenase 1 [Source:HGNC Symbol;Acc:HGNC:6970] | 271 | 0.772 | 0.1295 | Yes |
| 12 | ACO2 | aconitase 2 [Source:HGNC Symbol;Acc:HGNC:118] | 290 | 0.756 | 0.1403 | Yes |
| 13 | TREX1 | three prime repair exonuclease 1 [Source:HGNC Symbol;Acc:HGNC:12269] | 393 | 0.709 | 0.1437 | Yes |
| 14 | UQCRC1 | ubiquinol-cytochrome c reductase core protein 1 [Source:HGNC Symbol;Acc:HGNC:12585] | 473 | 0.672 | 0.1482 | Yes |
| 15 | NDUFB3 | NADH:ubiquinone oxidoreductase subunit B3 [Source:HGNC Symbol;Acc:HGNC:7698] | 483 | 0.667 | 0.1582 | Yes |
| 16 | NDUFS4 | NADH:ubiquinone oxidoreductase subunit S4 [Source:HGNC Symbol;Acc:HGNC:7711] | 531 | 0.647 | 0.1649 | Yes |
| 17 | NDUFA3 | NADH:ubiquinone oxidoreductase subunit A3 [Source:HGNC Symbol;Acc:HGNC:7686] | 550 | 0.641 | 0.1738 | Yes |
| 18 | COQ9 | coenzyme Q9 [Source:HGNC Symbol;Acc:HGNC:25302] | 561 | 0.636 | 0.1833 | Yes |
| 19 | ATP5PD | ATP synthase peripheral stalk subunit d [Source:HGNC Symbol;Acc:HGNC:845] | 564 | 0.635 | 0.1933 | Yes |
| 20 | DLD | dihydrolipoamide dehydrogenase [Source:HGNC Symbol;Acc:HGNC:2898] | 565 | 0.635 | 0.2035 | Yes |
| 21 | COX5B | cytochrome c oxidase subunit 5B [Source:HGNC Symbol;Acc:HGNC:2269] | 585 | 0.628 | 0.2121 | Yes |
| 22 | MDH2 | malate dehydrogenase 2 [Source:HGNC Symbol;Acc:HGNC:6971] | 615 | 0.618 | 0.2197 | Yes |
| 23 | ANTKMT | adenine nucleotide translocase lysine methyltransferase [Source:HGNC Symbol;Acc:HGNC:14152] | 618 | 0.618 | 0.2295 | Yes |
| 24 | NDUFA4 | NDUFA4 mitochondrial complex associated [Source:HGNC Symbol;Acc:HGNC:7687] | 624 | 0.613 | 0.2390 | Yes |
| 25 | COX6A1 | cytochrome c oxidase subunit 6A1 [Source:HGNC Symbol;Acc:HGNC:2277] | 653 | 0.605 | 0.2465 | Yes |
| 26 | MSH2 | mutS homolog 2 [Source:HGNC Symbol;Acc:HGNC:7325] | 739 | 0.584 | 0.2492 | Yes |
| 27 | ATP5PO | ATP synthase peripheral stalk subunit OSCP [Source:HGNC Symbol;Acc:HGNC:850] | 840 | 0.558 | 0.2503 | Yes |
| 28 | NDUFA2 | NADH:ubiquinone oxidoreductase subunit A2 [Source:HGNC Symbol;Acc:HGNC:7685] | 878 | 0.549 | 0.2562 | Yes |
| 29 | NDUFB5 | NADH:ubiquinone oxidoreductase subunit B5 [Source:HGNC Symbol;Acc:HGNC:7700] | 902 | 0.542 | 0.2631 | Yes |
| 30 | NDUFC2 | NADH:ubiquinone oxidoreductase subunit C2 [Source:HGNC Symbol;Acc:HGNC:7706] | 929 | 0.532 | 0.2696 | Yes |
| 31 | NDUFA9 | NADH:ubiquinone oxidoreductase subunit A9 [Source:HGNC Symbol;Acc:HGNC:7693] | 943 | 0.529 | 0.2771 | Yes |
| 32 | DNAJC15 | DnaJ heat shock protein family (Hsp40) member C15 [Source:HGNC Symbol;Acc:HGNC:20325] | 954 | 0.527 | 0.2848 | Yes |
| 33 | IDH2 | isocitrate dehydrogenase (NADP(+)) 2 [Source:HGNC Symbol;Acc:HGNC:5383] | 967 | 0.525 | 0.2923 | Yes |
| 34 | NDUFB4 | NADH:ubiquinone oxidoreductase subunit B4 [Source:HGNC Symbol;Acc:HGNC:7699] | 968 | 0.525 | 0.3007 | Yes |
| 35 | UQCRQ | ubiquinol-cytochrome c reductase complex III subunit VII [Source:HGNC Symbol;Acc:HGNC:29594] | 986 | 0.521 | 0.3077 | Yes |
| 36 | NDUFB7 | NADH:ubiquinone oxidoreductase subunit B7 [Source:HGNC Symbol;Acc:HGNC:7702] | 1033 | 0.510 | 0.3123 | Yes |
| 37 | NDUFB1 | NADH:ubiquinone oxidoreductase subunit B1 [Source:HGNC Symbol;Acc:HGNC:7695] | 1055 | 0.506 | 0.3188 | Yes |
| 38 | NDUFS7 | NADH:ubiquinone oxidoreductase core subunit S7 [Source:HGNC Symbol;Acc:HGNC:7714] | 1071 | 0.501 | 0.3257 | Yes |
| 39 | NDUFC1 | NADH:ubiquinone oxidoreductase subunit C1 [Source:HGNC Symbol;Acc:HGNC:7705] | 1091 | 0.499 | 0.3322 | Yes |
| 40 | NDUFA7 | NADH:ubiquinone oxidoreductase subunit A7 [Source:HGNC Symbol;Acc:HGNC:7691] | 1097 | 0.496 | 0.3398 | Yes |
| 41 | ARL2 | ADP ribosylation factor like GTPase 2 [Source:HGNC Symbol;Acc:HGNC:693] | 1179 | 0.481 | 0.3411 | Yes |
| 42 | NDUFS8 | NADH:ubiquinone oxidoreductase core subunit S8 [Source:HGNC Symbol;Acc:HGNC:7715] | 1208 | 0.474 | 0.3465 | Yes |
| 43 | NDUFS1 | NADH:ubiquinone oxidoreductase core subunit S1 [Source:HGNC Symbol;Acc:HGNC:7707] | 1229 | 0.471 | 0.3525 | Yes |
| 44 | ETFB | electron transfer flavoprotein subunit beta [Source:HGNC Symbol;Acc:HGNC:3482] | 1289 | 0.460 | 0.3553 | Yes |
| 45 | ATP5PB | ATP synthase peripheral stalk-membrane subunit b [Source:HGNC Symbol;Acc:HGNC:840] | 1293 | 0.459 | 0.3624 | Yes |
| 46 | ATP5MF | ATP synthase membrane subunit f [Source:HGNC Symbol;Acc:HGNC:848] | 1330 | 0.453 | 0.3669 | Yes |
| 47 | PARK7 | Parkinsonism associated deglycase [Source:HGNC Symbol;Acc:HGNC:16369] | 1406 | 0.442 | 0.3681 | Yes |
| 48 | NDUFS6 | NADH:ubiquinone oxidoreductase subunit S6 [Source:HGNC Symbol;Acc:HGNC:7713] | 1417 | 0.441 | 0.3744 | Yes |
| 49 | SUCLG2 | succinate-CoA ligase GDP-forming subunit beta [Source:HGNC Symbol;Acc:HGNC:11450] | 1472 | 0.432 | 0.3771 | Yes |
| 50 | SHMT2 | serine hydroxymethyltransferase 2 [Source:HGNC Symbol;Acc:HGNC:10852] | 1518 | 0.426 | 0.3804 | Yes |
| 51 | ISCU | iron-sulfur cluster assembly enzyme [Source:HGNC Symbol;Acc:HGNC:29882] | 1530 | 0.423 | 0.3863 | Yes |
| 52 | NDUFV2 | NADH:ubiquinone oxidoreductase core subunit V2 [Source:HGNC Symbol;Acc:HGNC:7717] | 1642 | 0.407 | 0.3841 | Yes |
| 53 | NDUFV1 | NADH:ubiquinone oxidoreductase core subunit V1 [Source:HGNC Symbol;Acc:HGNC:7716] | 1663 | 0.404 | 0.3890 | Yes |
| 54 | CAT | catalase [Source:HGNC Symbol;Acc:HGNC:1516] | 1731 | 0.394 | 0.3901 | Yes |
| 55 | ETFA | electron transfer flavoprotein subunit alpha [Source:HGNC Symbol;Acc:HGNC:3481] | 1797 | 0.385 | 0.3912 | Yes |
| 56 | SNCA | synuclein alpha [Source:HGNC Symbol;Acc:HGNC:11138] | 1800 | 0.384 | 0.3972 | Yes |
| 57 | SDHB | succinate dehydrogenase complex iron sulfur subunit B [Source:HGNC Symbol;Acc:HGNC:10681] | 1818 | 0.382 | 0.4020 | Yes |
| 58 | STOML2 | stomatin like 2 [Source:HGNC Symbol;Acc:HGNC:14559] | 1819 | 0.382 | 0.4081 | Yes |
| 59 | SURF1 | SURF1 cytochrome c oxidase assembly factor [Source:HGNC Symbol;Acc:HGNC:11474] | 1829 | 0.381 | 0.4135 | Yes |
| 60 | NIPSNAP2 | nipsnap homolog 2 [Source:HGNC Symbol;Acc:HGNC:4179] | 1886 | 0.374 | 0.4151 | Yes |
| 61 | COX7A1 | cytochrome c oxidase subunit 7A1 [Source:HGNC Symbol;Acc:HGNC:2287] | 2045 | 0.356 | 0.4084 | Yes |
| 62 | PINK1 | PTEN induced kinase 1 [Source:HGNC Symbol;Acc:HGNC:14581] | 2097 | 0.350 | 0.4100 | Yes |
| 63 | TNF | tumor necrosis factor [Source:HGNC Symbol;Acc:HGNC:11892] | 2098 | 0.350 | 0.4157 | Yes |
| 64 | COX4I1 | cytochrome c oxidase subunit 4I1 [Source:HGNC Symbol;Acc:HGNC:2265] | 2167 | 0.342 | 0.4158 | Yes |
| 65 | ATP5F1C | ATP synthase F1 subunit gamma [Source:HGNC Symbol;Acc:HGNC:833] | 2190 | 0.339 | 0.4195 | Yes |
| 66 | IREB2 | iron responsive element binding protein 2 [Source:HGNC Symbol;Acc:HGNC:6115] | 2215 | 0.337 | 0.4231 | Yes |
| 67 | SDHA | succinate dehydrogenase complex flavoprotein subunit A [Source:HGNC Symbol;Acc:HGNC:10680] | 2231 | 0.335 | 0.4273 | Yes |
| 68 | NDUFS5 | NADH:ubiquinone oxidoreductase subunit S5 [Source:HGNC Symbol;Acc:HGNC:7712] | 2267 | 0.330 | 0.4298 | Yes |
| 69 | SUCLG1 | succinate-CoA ligase GDP/ADP-forming subunit alpha [Source:HGNC Symbol;Acc:HGNC:11449] | 2269 | 0.330 | 0.4350 | Yes |
| 70 | CCNB1 | cyclin B1 [Source:HGNC Symbol;Acc:HGNC:1579] | 2359 | 0.319 | 0.4332 | Yes |
| 71 | NNT | nicotinamide nucleotide transhydrogenase [Source:HGNC Symbol;Acc:HGNC:7863] | 2425 | 0.311 | 0.4331 | Yes |
| 72 | CHCHD2 | coiled-coil-helix-coiled-coil-helix domain containing 2 [Source:HGNC Symbol;Acc:HGNC:21645] | 2431 | 0.310 | 0.4377 | Yes |
| 73 | PDHA1 | pyruvate dehydrogenase E1 subunit alpha 1 [Source:HGNC Symbol;Acc:HGNC:8806] | 2433 | 0.310 | 0.4426 | Yes |
| 74 | IL10RB | interleukin 10 receptor subunit beta [Source:HGNC Symbol;Acc:HGNC:5965] | 2438 | 0.309 | 0.4472 | Yes |
| 75 | NDUFB2 | NADH:ubiquinone oxidoreductase subunit B2 [Source:HGNC Symbol;Acc:HGNC:7697] | 2460 | 0.307 | 0.4505 | Yes |
| 76 | COX7A2L | cytochrome c oxidase subunit 7A2 like [Source:HGNC Symbol;Acc:HGNC:2289] | 2517 | 0.302 | 0.4509 | Yes |
| 77 | NDUFB8 | NADH:ubiquinone oxidoreductase subunit B8 [Source:HGNC Symbol;Acc:HGNC:7703] | 2623 | 0.288 | 0.4473 | No |
| 78 | BLOC1S1 | biogenesis of lysosomal organelles complex 1 subunit 1 [Source:HGNC Symbol;Acc:HGNC:4200] | 2657 | 0.284 | 0.4493 | No |
| 79 | ATP5F1D | ATP synthase F1 subunit delta [Source:HGNC Symbol;Acc:HGNC:837] | 2701 | 0.279 | 0.4504 | No |
| 80 | ATP5MG | ATP synthase membrane subunit g [Source:HGNC Symbol;Acc:HGNC:14247] | 2941 | 0.256 | 0.4357 | No |
| 81 | OPN3 | opsin 3 [Source:HGNC Symbol;Acc:HGNC:14007] | 2961 | 0.254 | 0.4383 | No |
| 82 | ATP5F1B | ATP synthase F1 subunit beta [Source:HGNC Symbol;Acc:HGNC:830] | 3024 | 0.247 | 0.4374 | No |
| 83 | NDUFS2 | NADH:ubiquinone oxidoreductase core subunit S2 [Source:HGNC Symbol;Acc:HGNC:7708] | 3072 | 0.243 | 0.4376 | No |
| 84 | DHTKD1 | dehydrogenase E1 and transketolase domain containing 1 [Source:HGNC Symbol;Acc:HGNC:23537] | 3112 | 0.239 | 0.4384 | No |
| 85 | SDHC | succinate dehydrogenase complex subunit C [Source:HGNC Symbol;Acc:HGNC:10682] | 3145 | 0.235 | 0.4396 | No |
| 86 | COX7A2 | cytochrome c oxidase subunit 7A2 [Source:HGNC Symbol;Acc:HGNC:2288] | 3164 | 0.234 | 0.4420 | No |
| 87 | SUCLA2 | succinate-CoA ligase ADP-forming subunit beta [Source:HGNC Symbol;Acc:HGNC:11448] | 3228 | 0.228 | 0.4407 | No |
| 88 | GBA | glucosylceramidase beta [Source:HGNC Symbol;Acc:HGNC:4177] | 3327 | 0.219 | 0.4365 | No |
| 89 | VCP | valosin containing protein [Source:HGNC Symbol;Acc:HGNC:12666] | 3518 | 0.201 | 0.4248 | No |
| 90 | IFNAR1 | interferon alpha and beta receptor subunit 1 [Source:HGNC Symbol;Acc:HGNC:5432] | 3587 | 0.195 | 0.4226 | No |
| 91 | COX15 | cytochrome c oxidase assembly homolog COX15 [Source:HGNC Symbol;Acc:HGNC:2263] | 3727 | 0.183 | 0.4146 | No |
| 92 | CDK1 | cyclin dependent kinase 1 [Source:HGNC Symbol;Acc:HGNC:1722] | 3744 | 0.182 | 0.4163 | No |
| 93 | UQCRH | ubiquinol-cytochrome c reductase hinge protein [Source:HGNC Symbol;Acc:HGNC:12590] | 3827 | 0.174 | 0.4126 | No |
| 94 | NDUFA6 | NADH:ubiquinone oxidoreductase subunit A6 [Source:HGNC Symbol;Acc:HGNC:7690] | 3834 | 0.173 | 0.4149 | No |
| 95 | SIRT3 | sirtuin 3 [Source:HGNC Symbol;Acc:HGNC:14931] | 4009 | 0.155 | 0.4037 | No |
| 96 | UQCRC2 | ubiquinol-cytochrome c reductase core protein 2 [Source:HGNC Symbol;Acc:HGNC:12586] | 4020 | 0.154 | 0.4054 | No |
| 97 | SDHD | succinate dehydrogenase complex subunit D [Source:HGNC Symbol;Acc:HGNC:10683] | 4055 | 0.151 | 0.4052 | No |
| 98 | CISD1 | CDGSH iron sulfur domain 1 [Source:HGNC Symbol;Acc:HGNC:30880] | 4104 | 0.146 | 0.4037 | No |
| 99 | ATP5F1A | ATP synthase F1 subunit alpha [Source:HGNC Symbol;Acc:HGNC:823] | 4137 | 0.143 | 0.4035 | No |
| 100 | NDUFAF1 | NADH:ubiquinone oxidoreductase complex assembly factor 1 [Source:HGNC Symbol;Acc:HGNC:18828] | 4388 | 0.122 | 0.3858 | No |
| 101 | NDUFAB1 | NADH:ubiquinone oxidoreductase subunit AB1 [Source:HGNC Symbol;Acc:HGNC:7694] | 4445 | 0.118 | 0.3833 | No |
| 102 | TEFM | "transcription elongation factor, mitochondrial [Source:HGNC Symbol;Acc:HGNC:26223]" | 4447 | 0.117 | 0.3851 | No |
| 103 | IDH3G | isocitrate dehydrogenase (NAD(+)) 3 non-catalytic subunit gamma [Source:HGNC Symbol;Acc:HGNC:5386] | 4504 | 0.113 | 0.3825 | No |
| 104 | COX7C | cytochrome c oxidase subunit 7C [Source:HGNC Symbol;Acc:HGNC:2292] | 4539 | 0.110 | 0.3816 | No |
| 105 | ME3 | malic enzyme 3 [Source:HGNC Symbol;Acc:HGNC:6985] | 4613 | 0.104 | 0.3776 | No |
| 106 | TRAP1 | TNF receptor associated protein 1 [Source:HGNC Symbol;Acc:HGNC:16264] | 4756 | 0.090 | 0.3679 | No |
| 107 | ATP7A | ATPase copper transporting alpha [Source:HGNC Symbol;Acc:HGNC:869] | 4814 | 0.086 | 0.3648 | No |
| 108 | CYC1 | cytochrome c1 [Source:HGNC Symbol;Acc:HGNC:2579] | 4879 | 0.082 | 0.3610 | No |
| 109 | UQCRB | ubiquinol-cytochrome c reductase binding protein [Source:HGNC Symbol;Acc:HGNC:12582] | 5003 | 0.073 | 0.3525 | No |
| 110 | COX6A2 | cytochrome c oxidase subunit 6A2 [Source:HGNC Symbol;Acc:HGNC:2279] | 5242 | 0.053 | 0.3347 | No |
| 111 | COX7A2P2 | cytochrome c oxidase subunit 7A2 pseudogene 2 [Source:HGNC Symbol;Acc:HGNC:2290] | 5263 | 0.051 | 0.3339 | No |
| 112 | COX5A | cytochrome c oxidase subunit 5A [Source:HGNC Symbol;Acc:HGNC:2267] | 5329 | 0.045 | 0.3296 | No |
| 113 | SLC25A14 | solute carrier family 25 member 14 [Source:HGNC Symbol;Acc:HGNC:10984] | 5333 | 0.045 | 0.3300 | No |
| 114 | IDE | insulin degrading enzyme [Source:HGNC Symbol;Acc:HGNC:5381] | 5491 | 0.033 | 0.3182 | No |
| 115 | BNIP3 | BCL2 interacting protein 3 [Source:HGNC Symbol;Acc:HGNC:1084] | 5501 | 0.032 | 0.3180 | No |
| 116 | SLC25A12 | solute carrier family 25 member 12 [Source:HGNC Symbol;Acc:HGNC:10982] | 5506 | 0.032 | 0.3182 | No |
| 117 | UQCRFS1 | "ubiquinol-cytochrome c reductase, Rieske iron-sulfur polypeptide 1 [Source:HGNC Symbol;Acc:HGNC:12587]" | 5509 | 0.032 | 0.3186 | No |
| 118 | NUPR1 | "nuclear protein 1, transcriptional regulator [Source:HGNC Symbol;Acc:HGNC:29990]" | 5616 | 0.023 | 0.3106 | No |
| 119 | DLAT | dihydrolipoamide S-acetyltransferase [Source:HGNC Symbol;Acc:HGNC:2896] | 5647 | 0.020 | 0.3086 | No |
| 120 | ADSL | adenylosuccinate lyase [Source:HGNC Symbol;Acc:HGNC:291] | 5685 | 0.017 | 0.3059 | No |
| 121 | BID | BH3 interacting domain death agonist [Source:HGNC Symbol;Acc:HGNC:1050] | 5715 | 0.014 | 0.3039 | No |
| 122 | FH | fumarate hydratase [Source:HGNC Symbol;Acc:HGNC:3700] | 5729 | 0.013 | 0.3031 | No |
| 123 | POLG2 | "DNA polymerase gamma 2, accessory subunit [Source:HGNC Symbol;Acc:HGNC:9180]" | 5892 | 0.003 | 0.2904 | No |
| 124 | CBFA2T3 | CBFA2/RUNX1 partner transcriptional co-repressor 3 [Source:HGNC Symbol;Acc:HGNC:1537] | 5951 | -0.001 | 0.2859 | No |
| 125 | OXA1L | OXA1L mitochondrial inner membrane protein [Source:HGNC Symbol;Acc:HGNC:8526] | 5974 | -0.003 | 0.2842 | No |
| 126 | COX10 | cytochrome c oxidase assembly factor heme A:farnesyltransferase COX10 [Source:HGNC Symbol;Acc:HGNC:2260] | 6092 | -0.012 | 0.2752 | No |
| 127 | ATP5PF | ATP synthase peripheral stalk subunit F6 [Source:HGNC Symbol;Acc:HGNC:847] | 6109 | -0.013 | 0.2741 | No |
| 128 | NOP53 | NOP53 ribosome biogenesis factor [Source:HGNC Symbol;Acc:HGNC:4333] | 6334 | -0.031 | 0.2570 | No |
| 129 | ATP5ME | ATP synthase membrane subunit e [Source:HGNC Symbol;Acc:HGNC:846] | 6365 | -0.032 | 0.2552 | No |
| 130 | ATP5F1E | ATP synthase F1 subunit epsilon [Source:HGNC Symbol;Acc:HGNC:838] | 6405 | -0.036 | 0.2527 | No |
| 131 | IFNG | interferon gamma [Source:HGNC Symbol;Acc:HGNC:5438] | 6410 | -0.036 | 0.2529 | No |
| 132 | IL4 | interleukin 4 [Source:HGNC Symbol;Acc:HGNC:6014] | 6673 | -0.054 | 0.2332 | No |
| 133 | SLC25A23 | solute carrier family 25 member 23 [Source:HGNC Symbol;Acc:HGNC:19375] | 6676 | -0.054 | 0.2339 | No |
| 134 | NDUFA5 | NADH:ubiquinone oxidoreductase subunit A5 [Source:HGNC Symbol;Acc:HGNC:7688] | 6789 | -0.062 | 0.2261 | No |
| 135 | MTFR1 | mitochondrial fission regulator 1 [Source:HGNC Symbol;Acc:HGNC:29510] | 6890 | -0.071 | 0.2194 | No |
| 136 | IDH1 | isocitrate dehydrogenase (NADP(+)) 1 [Source:HGNC Symbol;Acc:HGNC:5382] | 7109 | -0.089 | 0.2037 | No |
| 137 | GPD2 | glycerol-3-phosphate dehydrogenase 2 [Source:HGNC Symbol;Acc:HGNC:4456] | 7206 | -0.097 | 0.1977 | No |
| 138 | GPD1 | glycerol-3-phosphate dehydrogenase 1 [Source:HGNC Symbol;Acc:HGNC:4455] | 7420 | -0.112 | 0.1828 | No |
| 139 | ACTN3 | actinin alpha 3 [Source:HGNC Symbol;Acc:HGNC:165] | 7557 | -0.124 | 0.1741 | No |
| 140 | PIK3CA | "phosphatidylinositol-4,5-bisphosphate 3-kinase catalytic subunit alpha [Source:HGNC Symbol;Acc:HGNC:8975]" | 7733 | -0.136 | 0.1625 | No |
| 141 | TRPV4 | transient receptor potential cation channel subfamily V member 4 [Source:HGNC Symbol;Acc:HGNC:18083] | 7925 | -0.149 | 0.1499 | No |
| 142 | FXN | frataxin [Source:HGNC Symbol;Acc:HGNC:3951] | 8057 | -0.160 | 0.1422 | No |
| 143 | PDE12 | phosphodiesterase 12 [Source:HGNC Symbol;Acc:HGNC:25386] | 8306 | -0.178 | 0.1255 | No |
| 144 | CYCS | "cytochrome c, somatic [Source:HGNC Symbol;Acc:HGNC:19986]" | 8410 | -0.186 | 0.1204 | No |
| 145 | OGDH | oxoglutarate dehydrogenase [Source:HGNC Symbol;Acc:HGNC:8124] | 8503 | -0.194 | 0.1163 | No |
| 146 | RHOA | ras homolog family member A [Source:HGNC Symbol;Acc:HGNC:667] | 8601 | -0.201 | 0.1119 | No |
| 147 | SLC25A22 | solute carrier family 25 member 22 [Source:HGNC Symbol;Acc:HGNC:19954] | 8682 | -0.208 | 0.1090 | No |
| 148 | SLC25A13 | solute carrier family 25 member 13 [Source:HGNC Symbol;Acc:HGNC:10983] | 8713 | -0.210 | 0.1100 | No |
| 149 | FLCN | folliculin [Source:HGNC Symbol;Acc:HGNC:27310] | 8848 | -0.221 | 0.1030 | No |
| 150 | ABCD1 | ATP binding cassette subfamily D member 1 [Source:HGNC Symbol;Acc:HGNC:61] | 8930 | -0.226 | 0.1003 | No |
| 151 | PDHA2 | pyruvate dehydrogenase E1 subunit alpha 2 [Source:HGNC Symbol;Acc:HGNC:8807] | 9124 | -0.241 | 0.0890 | No |
| 152 | ACO1 | aconitase 1 [Source:HGNC Symbol;Acc:HGNC:117] | 9426 | -0.266 | 0.0696 | No |
| 153 | AK4 | adenylate kinase 4 [Source:HGNC Symbol;Acc:HGNC:363] | 9682 | -0.290 | 0.0542 | No |
| 154 | MLXIPL | MLX interacting protein like [Source:HGNC Symbol;Acc:HGNC:12744] | 9882 | -0.310 | 0.0436 | No |
| 155 | PPIF | peptidylprolyl isomerase F [Source:HGNC Symbol;Acc:HGNC:9259] | 9999 | -0.321 | 0.0396 | No |
| 156 | COQ10B | coenzyme Q10B [Source:HGNC Symbol;Acc:HGNC:25819] | 10099 | -0.331 | 0.0371 | No |
| 157 | ETFDH | electron transfer flavoprotein dehydrogenase [Source:HGNC Symbol;Acc:HGNC:3483] | 10203 | -0.341 | 0.0345 | No |
| 158 | PRDM16 | PR/SET domain 16 [Source:HGNC Symbol;Acc:HGNC:14000] | 10211 | -0.342 | 0.0395 | No |
| 159 | IDH3A | isocitrate dehydrogenase (NAD(+)) 3 catalytic subunit alpha [Source:HGNC Symbol;Acc:HGNC:5384] | 10426 | -0.364 | 0.0285 | No |
| 160 | TAFAZZIN | "tafazzin, phospholipid-lysophospholipid transacylase [Source:HGNC Symbol;Acc:HGNC:11577]" | 10429 | -0.364 | 0.0342 | No |
| 161 | PPARGC1A | PPARG coactivator 1 alpha [Source:HGNC Symbol;Acc:HGNC:9237] | 10504 | -0.374 | 0.0344 | No |
| 162 | DLST | dihydrolipoamide S-succinyltransferase [Source:HGNC Symbol;Acc:HGNC:2911] | 10634 | -0.386 | 0.0304 | No |
| 163 | CS | citrate synthase [Source:HGNC Symbol;Acc:HGNC:2422] | 10748 | -0.399 | 0.0279 | No |
| 164 | OGDHL | oxoglutarate dehydrogenase L [Source:HGNC Symbol;Acc:HGNC:25590] | 10754 | -0.399 | 0.0340 | No |
| 165 | PLEC | plectin [Source:HGNC Symbol;Acc:HGNC:9069] | 10799 | -0.405 | 0.0370 | No |
| 166 | MYBBP1A | MYB binding protein 1a [Source:HGNC Symbol;Acc:HGNC:7546] | 11188 | -0.456 | 0.0138 | No |
| 167 | IDH3B | isocitrate dehydrogenase (NAD(+)) 3 non-catalytic subunit beta [Source:HGNC Symbol;Acc:HGNC:5385] | 11304 | -0.472 | 0.0124 | No |
| 168 | NFATC4 | nuclear factor of activated T cells 4 [Source:HGNC Symbol;Acc:HGNC:7778] | 11363 | -0.481 | 0.0155 | No |
| 169 | SCO2 | synthesis of cytochrome C oxidase 2 [Source:HGNC Symbol;Acc:HGNC:10604] | 11410 | -0.488 | 0.0198 | No |
| 170 | CYP1A2 | cytochrome P450 family 1 subfamily A member 2 [Source:HGNC Symbol;Acc:HGNC:2596] | 11719 | -0.536 | 0.0042 | No |
| 171 | HIF1A | hypoxia inducible factor 1 subunit alpha [Source:HGNC Symbol;Acc:HGNC:4910] | 11833 | -0.556 | 0.0042 | No |
| 172 | NOS2 | nitric oxide synthase 2 [Source:HGNC Symbol;Acc:HGNC:7873] | 12262 | -0.665 | -0.0187 | No |
| 173 | UCN | urocortin [Source:HGNC Symbol;Acc:HGNC:12516] | 12536 | -0.765 | -0.0279 | No |
| 174 | NR4A3 | nuclear receptor subfamily 4 group A member 3 [Source:HGNC Symbol;Acc:HGNC:7982] | 12543 | -0.769 | -0.0160 | No |
| 175 | SOD2 | superoxide dismutase 2 [Source:HGNC Symbol;Acc:HGNC:11180] | 12685 | -0.853 | -0.0134 | No |
| 176 | MT-ND5 | mitochondrially encoded NADH:ubiquinone oxidoreductase core subunit 5 [Source:HGNC Symbol;Acc:HGNC:7461] | 12743 | -0.906 | -0.0033 | No |
| 177 | MYC | "MYC proto-oncogene, bHLH transcription factor [Source:HGNC Symbol;Acc:HGNC:7553]" | 12801 | -0.970 | 0.0078 | No |
Table: GSEA details [plain text format]

  

Fig 2: GOBP\_CELLULAR\_RESPIRATION      
 Blue-Pink O' Gram in the Space of the Analyzed GeneSet

  

Fig 3: GOBP\_CELLULAR\_RESPIRATION: Random ES distribution      
 Gene set null distribution of ES for **GOBP\_CELLULAR\_RESPIRATION**
